# Snagger: A user-friendly program for incorporating additional information for tagSNP selection

**DOI:** 10.1186/1471-2105-9-174

**Published:** 2008-03-27

**Authors:** Christopher K Edlund, Won H Lee, Dalin Li, David J Van Den Berg, David V Conti

**Affiliations:** 1 Keck School of Medicine, University of Southern California, Los Angeles, California 90033, USA; 2 Department of Preventive Medicine, Keck School of Medicine, University of Southern California, Los Angeles, California 90033, USA; 3 Zilkha Neurogenetic Institute, University of Southern California, Los Angeles, CA 90033, USA

## Abstract

**Background:**

There has been considerable effort focused on developing efficient programs for tagging single-nucleotide polymorphisms (SNPs). Many of these programs do not account for potential reduced genomic coverage resulting from genotyping failures nor do they preferentially select SNPs based on functionality, which may be more likely to be biologically important.

**Results:**

We have developed a user-friendly and efficient software program, Snagger, as an extension to the existing open-source software, Haploview, which uses pairwise *r*^2 ^linkage disequilibrium between single nucleotide polymorphisms (SNPs) to select tagSNPs. Snagger distinguishes itself from existing SNP selection algorithms, including Tagger, by providing user options that allow for: (1) prioritization of tagSNPs based on certain characteristics, including platform-specific design scores, functionality (i.e., coding status), and chromosomal position, (2) efficient selection of SNPs across multiple populations, (3) selection of tagSNPs outside defined genomic regions to improve coverage and genotyping success, and (4) picking of surrogate tagSNPs that serve as backups for tagSNPs whose failure would result in a significant loss of data. Using HapMap genotype data from ten ENCODE regions and design scores for the Illumina platform, we show similar coverage and design score distribution and fewer total tagSNPs selected by Snagger compared to the web server Tagger.

**Conclusion:**

Snagger improves upon current available tagSNP software packages by providing a means for researchers to select tagSNPs that reliably capture genetic variation across multiple populations while accounting for significant genotyping failure risk and prioritizing on SNP-specific characteristics.

## Background

There has been extensive effort to develop and implement strategies for efficient selection of single nucleotide polymorphisms (SNPs) in candidate-gene association studies of complex disease. Due to the prohibitively high cost associated with genotyping every SNP within a given set of genes, methods have been developed to find a subset of these SNPs that capture the same genetic diversity. One of these methods includes a preliminary stage of genotyping in which linkage disequilibrium (LD) or haplotype block structure is estimated by genotyping a set of evenly distributed SNPs across one or more genes for a sample set representative of a given population. Two freely available software applications of note exist to facilitate this preliminary stage [1,2]. SNPHunter automates the filtering and selection of SNPs for genotyping, allowing the user to incorporate desired characteristics, such as chromosomal position and functionality [1]. Once genotyped, htSNPer1.0 can be used to define haplotype boundaries and select haplotype tagging SNPs (htSNPs) to capture underlying LD [2]. Using the resulting LD or haplotype information obtained from a first-stage sample, a second stage of genotyping for a smaller set of non-redundant SNPs is typically performed in a larger sample. For example, Haiman et al. demonstrated the use of a small multiethnic first-stage sample with dense genotyping in order to capture the genetic diversity within *CYP19*. Subsequent haplotype tagging SNPs were then genotyped in a larger case-control second-stage sample examining the association with breast cancer [3].

Recently, it has become common to use the publicly available HapMap database in place of the first stage of genotyping. HapMap, containing genotypes of 270 individuals in four geographically diverse populations for over three million SNPs, has become a reliable source for describing genetic diversity and inferring LD patterns in a target sample population [4,5]. Population genetic studies of underlying LD patterns have demonstrated that data from the HapMap project is sufficient in describing the underlying LD structure across multiple populations [6].

Once genotypes for a set of SNPs is obtained for a representative sample (either from a primary stage of genotyping or the publicly available HapMap database), two approaches can be used to select a minimal set of SNPs to be genotyped in a larger sample: "block-based" and "block-free" [7]. Block-based approaches use haplotype block structure and haplotype frequencies in order to select an informative, non-redundant, minimal set of SNPs that captures the underlying haplotype diversity [8,9]. Block-free approaches do not require this underlying block structure, and instead use pairwise LD between SNPs in order to select a minimal set of tagSNPs that capture all other SNPs at a defined threshold [10]. Block-based approaches have an advantage in that the possible interaction of a group of SNPs that are genetically linked can be measured as a haplotype. In a block-free approach, there is no guarantee that the selected tagSNPs will allow differentiation of haplotypes. However, a drawback of block-based approaches is that they only sample a fraction of the genetic diversity in regions with poor block structure. While both approaches offer advantages, we have focused on developing methods and tools for block-free approaches and we limit our comparison to Tagger [11], which implements a block-free algorithm.

There are several algorithms using block-free approaches to select tagSNPs [7,10,12-15]. Some of these algorithms are based on *D' *as a measure of LD [9,12], but the majority use *r*^2 ^[7,10,13-15], as it is a direct measure of association between SNPs [14] and inversely related to statistical power [10,14,16].

The current accessible algorithms, including the commonly used program Tagger [11], have some notable limitations. Some programs enable the user to forcibly include SNPs having *a priori *importance, such as known functionality [1,7,10,11,14,15], yet they lack the ability to prioritize additional tagSNP picking based on SNP features such as coding status or genomic location. Tagger [11] can consider design scores on a high-throughput genotyping platform when prioritizing tagSNPs, but does not take a SNP's probability of typing failure into account when tagging, nor does it allow SNPs outside of a targeted genomic region to be picked. Recently a few programs have been developed to allow for optimal selection of tagSNPs across multiple populations [17-19], yet they fail to incorporate one or more of the aforementioned features.

Typically, the set of possible tagSNPs in candidate gene studies using a block-free approach is limited to those SNPs which are located within the targeted genomic regions. However, patterns of LD can extend beyond the boundaries of these regions and are often non-contiguous when observing pairwise *r*^2 ^values between SNPs. This means that a SNP located outside of a targeted region may have a significantly high *r*^2 ^value with one or more SNPs located within the region, even if SNPs located between them are not in LD. Expanding the set of potential tagSNPs to include SNPs from outside a targeted region allows SNPs with higher probabilities of genotyping success to picked and increases the chance that SNPs unable to be genotyped will be captured.

In this paper we present a user-friendly and efficient block-free tagSNP selection program, Snagger, which improves upon current available SNP tagging algorithms and is available as an extension to Haploview. Our program allows the user to: (1) prioritize tagSNPs based on certain characteristics, including platform-specific design scores, functionality (i.e., coding status), and chromosomal position, (2) select tagSNPs across multiple populations, (3) select tagSNPs outside defined genomic regions to improve coverage and genotyping success, and (4) pick surrogate tagSNPs that serve as backups for tagSNPs whose failure would result in a significant loss of data. While many SNP selection programs and algorithms are designed to pick the minimal set of tagSNPs that will capture the underlying genetic structure, Snagger is designed to pick a set of tagSNPs that will capture the structure while also fulfilling user-defined characteristics and ensuring the best chance for genotyping success.

## Implementation

Snagger was implemented in Java as an extension to the existing open-source software, Haploview (version 3.3). It builds upon Haploview's user interface and uses its ability to import and filter genotype data in HapMap format and calculate pairwise LD metrics (*D' *and *r*^2^) between SNPs. In addition, it imports a score file containing design scores (e.g., from Illumina) and other relevant annotations for the SNPs in a defined genomic region.

### Data selection and filtering

**Figure 1:**
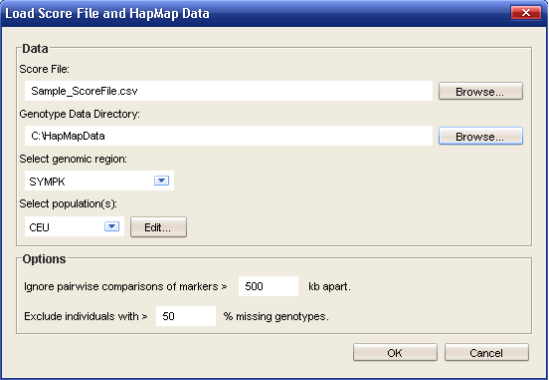
**Data input window**. Screen capture of the window where a user specifies a score file containing SNP information and design scores as well as the location of HapMap-formatted data. The user can select the genomic region and population(s) to load into Snagger here. In addition, a minimum pairwise comparison distance and minimum genotype percentage for individuals can be chosen.

**Figure 2:**
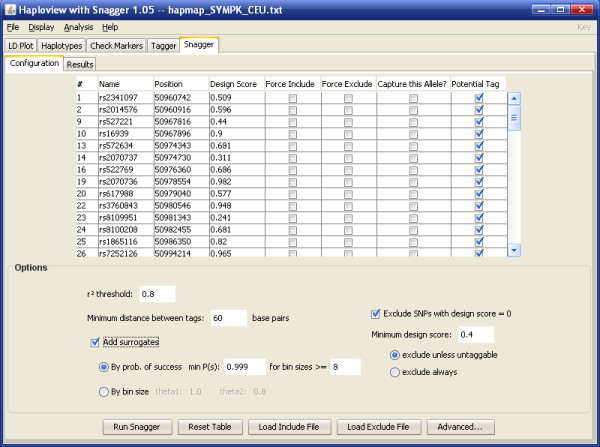
**Snagger tab in Haploview**. Screen capture of an additional tab in Haploview containing the Snagger program. Similar to the Tagger tab, a user can select various tagging and filtering parameters.

The user can specify a genomic region and ethnic group of interest (Figure 1) for tagSNP selection. Those SNPs passing user-defined filters, such as a minimum minor allele frequency (MAF), make up the set of SNPs, S = {s_1_, s_2_,..., s_m_}. Once data is imported, Haploview generates a table of all possible pairwise *r*^2 ^values between s_i _and s_j _(where i,j ∈ {1,..., m} and i ≠ j). Parameters and specifications for tagSNP selection can be specified by the user (Figure 2), including a minimum *r*^2 ^threshold, *r*^2^_min_, when determining the desired LD threshold between SNPs. A set of force-included, I = {i_1_, i_2_,..., i_n_}, or force-excluded, E = {e_1_, e_2_,..., e_o_}, SNPs within S can be inputted manually or imported as a separate file.

An option is provided for the user to enforce a minimum design score for tagSNPs as well as a minimum physical distance (in base pairs) between any two tagSNPs.

### Tagging algorithm

**Figure 3:**
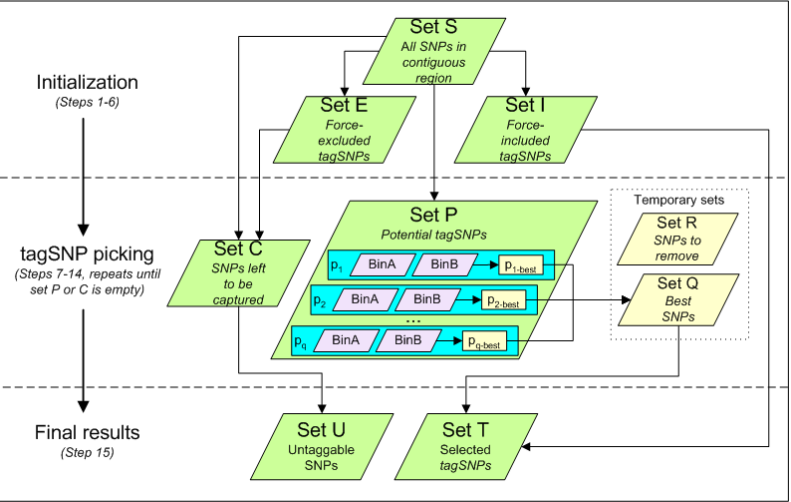
**Tagging algorithm overview**. High-level overview of set relations and stages of the Snagger tagging algorithm. Descriptions of set identifiers and step numbers are found in the Appendix.

Snagger allocates SNPs in set S into three primary sets for use in selecting tagSNPs (see Appendix and Figure 3 for a summary of the algorithm):

- C = {c_1_, c_2_,..., c_p_}, the set of all SNPs to be captured (i.e., "tagged")

- P = {p_1_, p_2_,..., p_q_}, the set of potential tagSNPs

- T = {t_1_, t_2_,..., t_r_}, the set of tagSNPs

Initially, all SNPs in set S are added to set P, and all SNPs within the region of interest and in set S are added to set C. If a set of force-included and/or force-excluded SNPs are specified, all force-included SNPs, set I, are added to set T, and SNPs in LD with set I are removed from set C. Snagger then generates the set of potential tagSNPs, set P, by adding all SNPs in set S except those SNPs in either set I or set E.

A SNP Score is assigned to each potential tagSNP in set P. It is a function of the SNP's probability of genotyping success (Pr [*GS*_*m*_]), MAF (*MAF*_*m*_), functionality (*Type*_*m*_, e.g., synonymous, nonsynonymous), and chromosomal position (*Loc*_*m*_, e.g., exon, intron).

*SS *= {*W*_GS _* Pr [*GS*_*m*_]} + {*W*_MAF _* *MAF*_*m*_} + {*W*_T _* *Type*_*m*_} + {*W*_L _* *Loc*_*m*_}

The probability of genotyping success, Pr [*GS*_*m*_], is calculated as a function of a SNP's design score.

$$\mathrm{Pr}\left[GS_{m}\right]=\frac{\exp(-0.27+3.78*DesignScore_{m})}{1+\exp(-0.27+3.78*DesignScore_{m})}$$

The default parameters for this function were estimated from modeling of failure rates as a function of Illumina design scores using data on 5,848 SNPs genotyped by the University of Southern California Genomics Core Facility. However, analogous scores from other platforms can be used to calculate this probability. The parameters can be changed using the software's interface, provided the user has the estimated parameters for the desired platform.

*MAF*_*m *_is a function of a SNP's MAF across the populations for which the user wishes to select tagSNPs and a user-defined *idealMAF*. Users can select from any of the four HapMap populations, import custom population data, or combine multiple populations into one. The default value for *idealMAF *is 0.5.

$$\begin{array}{l} MAF_{m}=\frac{\sum_{h=1}^{Hm}MAF_{mh}}{Hm}\\ MAF_{mh}=1-\frac{abs(idealMAF-observedMAF_{mh})}{0.5} \end{array}$$

For each SNP *m *having an observed MAF in a given population *h*, a MAF Score for that population, *MAF*_*mh*_, is calculated. The population-specific MAF Scores are then averaged across *H*_*m*_, the number of populations with an observed MAF for SNP *m*. SNPs having MAFs nearer to the ideal MAF will have MAF Scores closer to 1.

For SNP functionality and chromosomal position, the user can define values between 0 and 1 for specific characteristics (e.g., a SNP located in an exon leading to a nonsynonymous mutation, or a SNP located in an intron).

Weights (*W*_*GS*_, *W*_*MAF*_, *W*_*T*_, *W*_*L*_) are applied to each parameter, and can be modified by the user. If desired, the user can preferentially weight parameters so that tagSNPs having specific characteristics are more likely to be selected. The default value for each weight is zero except for the probability of genotyping success weight, where the default value is one. Thus, without user-specified weightings across parameters, SNP Scores only rely on genotyping success.

For each SNP in set P, a BinA is created containing those SNPs in C for which it can serve as a proxy. The LD threshold, *r*^2^_*min*_, is used as the entry criteria into respective BinAs.

A secondary bin, BinB, is created for each SNP *p_i _*in set P, containing only those SNPs that meet the *r*^2^_min _requirement with the SNP of interest and every other SNP in its BinB. The BinB is formed by first sorting the SNPs in* p_i_*'s BinA by the size of their BinA bins (highest to lowest). Initially, *p_i _*is added to its own BinB. Then, each SNP in* p_i_*'s BinA is sequentially added to its BinB if it is contained in the BinA of every SNP currently in* p_i_*'s BinB.

TagSNP picking starts by selecting the SNP from each BinB with the highest SNP Score and adding it to a temporary set, Q. From this narrowed set of potential tagSNPs, the SNP with the most SNPs in its respective BinA, t, is picked as a tagSNP and added to the set T. This tagSNP is removed from every BinB as well as the set of potential tagSNPs, set P. All SNPs in LD with t are removed from every BinA and the remaining set of SNPs to be captured, set C. This algorithm is repeated until either set C or set P become empty. If set P is empty, but set C is not, any SNPs remaining in set C are marked as "untaggable". Singletons are handled in the same way in the selection process, but become untaggable (i.e., uncaptured) if they violate a minimum tagSNP distance or design score requirement.

### Multiple populations

**Figure 4:**
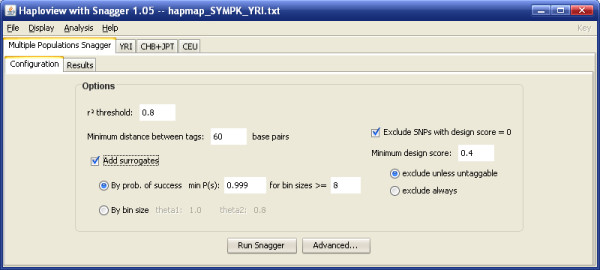
**Multiple Populations Snagger tab**. Screen capture of the main tab where a user specifies various tagging and filtering parameters used in the selection of tagSNPs across multiple populations.

**Figure 5:**
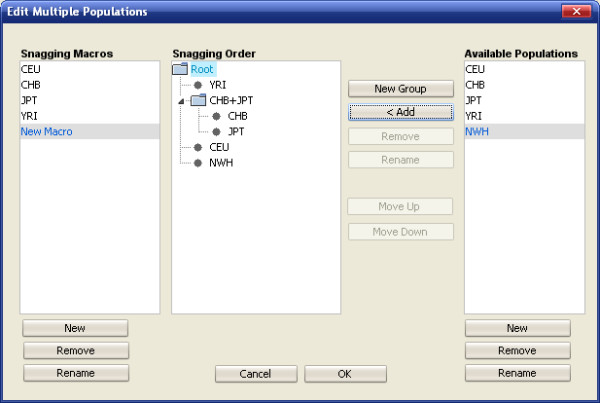
**Multiple populations editor window**. Screen capture of the window where a user can specify the order in which tagSNPs are selected across multiple populations.

Snagger has the ability to select tagSNPs across multiple ethnic groups. Using a user-defined order, it sequentially picks tagSNPs from the first population, using *r*^2^, design score, and surrogate picking parameter specifications (Figure 4), and forces them into the next population (Figure 5). This task is repeated for each population until the last population has been tagged. Since not all groups will share the same set of SNPs to capture (either due to filtering criteria or unavailability of genotypes), the final list of tagSNPs is the union of all tagSNPs in each group.

### Surrogate tagSNP picking

An inherent problem of any tagSNP approach is the possible loss of significant data if a tagSNP that predicts many SNPs fails genotyping. Although similar issues have been addressed in robust tagging software for block-based methods [20], block-free methods require a different solution. Two methods for adding surrogate tagSNPs are available in the software, including one based on probability of genotyping success and one based on the number of SNPs tagged.

The first method uses the probability of genotyping success, Pr [*GS*_*m*_] (Figure 2). As tagSNPs are chosen, every SNP receives a calculated probability of success (*CPS*) that is derived from the Pr [*GS*] of all chosen tagSNPs that can act as a proxy for that SNP. Given a SNP *m *with *n *tagSNPs:

*CPS*(*m*) = Pr [*GS*_1_] ∪ Pr [*GS*_2_] ∪...∪ Pr [*GS*_*n*_]

The user can enforce a minimum *CPS *on all SNPs whose corresponding tagSNPs capture a minimum number of SNPs. With this method, a SNP is not considered captured until its *CPS *meets or exceeds the user-defined threshold, its tagSNP's "captured" number is less than the cut-off, or there are no surrogates available. The software provides default values of 0.999 and 8 for the minimum *CPS *and minimum "captured" number of SNPs, respectively. The possible values for Pr [*GS*_*m*_] when using default parameters and Illumina design scores range from 0.43 to 0.98. It follows that any tagSNP predicting at least eight SNPs will at minimum require one surrogate tagSNP in order to achieve a *CPS *of 0.999 for the predicted SNPs. If either the tagSNP or surrogate has a sufficiently low design score that the threshold is not met, more surrogates will be selected as long as they are available. The default values are chosen so as any SNP in a large bin will have only one chance in a thousand that all the tagSNPs predicting it will fail.

The second method relies on a function that gives the required number of surrogates based on the how many SNPs a tagSNP is tagging (Figure 2):

*T *= log(Θ_1 _× *M*^Θ_2_) - 1,

where T is the number of surrogates needed and M is the number of SNPs tagged by a tagSNP, with the Θ_1 _and Θ_2 _values specified by the user. Every time a tagSNP is chosen, the above function is evaluated to check if and how many surrogates should be added. The surrogates are chosen from the tagSNP's BinB and added to the list of tagSNPs.

## Results

### SNP Score impact

Snagger's preferential selection of tagSNPs was evaluated using HapMap Public Release 21a genotype data [21] for 60 CEPH (Utah residents with ancestry from northern and western Europe) founder samples in the following 10 ENCODE regions: ENm010, ENm013, ENm014, ENr112, ENr113, ENr123, ENr131, ENr213, ENr232, ENr321.

For simulation purposes, we randomly marked one-eighth of all potential tagSNPs (936 of 7,479) as located in a coding region of the chromosome, which in practice would include both synonymous and nonsynonymous SNPs. The remaining 6,543 SNPs were marked as non-coding. In calculating the SNP Score, SNPs in the coding region received a weight of 1 and all other parameters had a weight of 0. Snagger selected 1,323 tagSNPs, of which 457 (34.5%) were "coding" SNPs. Favorable weighting for coding SNPs increased the proportion of tagSNPs located in a coding region nearly three times (from 12.5% of all potential tagSNPs to 34.5% of selected tagSNPs), and of all potential coding region tagSNPs, nearly half were selected (457 of 936).

**Figure 6:**
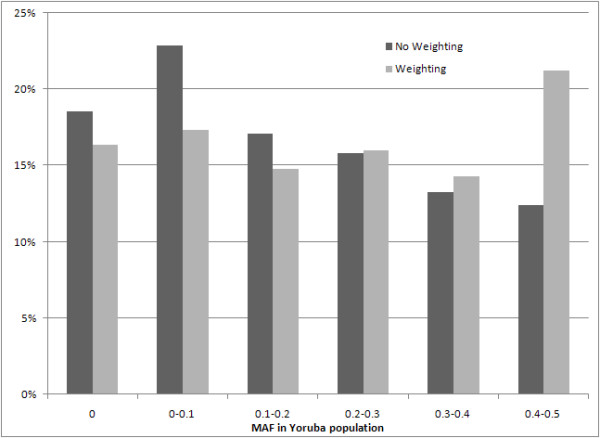
**Impact of SNP Score MAF Weighting on Average MAF**. SNP MAF distributions of the HapMap Yoruba population for tagSNPs chosen for only the CEPH population across 10 ENCODE regions, using two different scoring parameters. The first does not weigh on Yoruba MAF ("No Weighting"), the second does weigh ("Weighting").

We also compared tagSNPs selected for the HapMap CEPH population across 10 ENCODE regions, using a SNP Score with preferential weighting of MAF in the HapMap Yoruba (in Ibadan, Nigeria) population to no weighting at all. This was done in order to demonstrate the ability of Snagger to preferentially pick tagSNPs in one population (e.g., CEPH) which are common in another (e.g., Yoruba), such that resulting genotypes could potentially be compared in the future to the ungenotyped population. An MAF weight of 1 (for both CEPH and Yoruba populations) on the SNP Score and weights of 0 on all other parameters resulted in 21 percent of picked tagSNPs having an MAF between 0.4 and 0.5 in the Yoruba population. When not weighing on Yoruba MAF, only 12 percent of picked tagSNPs had an MAF between 0.4 and 0.5 in the Yoruba population. Also of note is the reduction in monomorphic tagSNPs in the Yoruba population from 19% with no weighting to 16% with weighting. The distribution of Yoruba MAFs for the chosen tagSNPs is shown in Figure 6.

### Comparison to Tagger

The efficiency and coverage of tagSNPs selected by Snagger and the web server Tagger were compared using the same HapMap SNP data and ENCODE regions (Table 1). Design scores were obtained for all SNPs, and used to compare the genotyping reliability of tagSNPs chosen by Snagger and the web server Tagger. Identical parameters were used in identifying potential tagSNPs to ensure comparability between the two software programs.

**Table 1:** Comparison of Tagger and Snagger. Comparison across 10 ENCODE regions between the web server Tagger and Snagger for: (a) the total of tagSNPs selected, (b) the average design score for selected tagSNPs, and (c) the percent coverage of the chromosome using an LD threshold of r^2 ^≥ 0.8.

	a. Total Number of tagSNPs		b. Average Design Score		c. Chromosomal Coverage at Maximum r^2 ^= 0.8	
ENCODE Region	Tagger	Snagger	Tagger	Snagger	Tagger	Snagger

ENm010	137	128	0.846	0.851	0.977	0.977
ENm013	107	82	0.953	0.972	0.992	0.996
ENm014	155	129	0.985	0.991	0.993	0.995
ENr112	192	162	1.035	1.029	0.996	0.998
ENr113	167	143	0.947	0.969	0.994	0.994
ENr123	193	139	0.846	0.858	0.989	0.993
ENr131	215	192	1.044	1.046	0.995	0.993
ENr213	129	112	1.024	1.033	0.994	0.992
ENr232	127	120	0.998	1.006	0.978	0.978
ENr321	122	114	1.009	1.022	0.987	0.988

In terms of the number of tagSNPs selected, Snagger was more efficient than the web server Tagger. Across every ENCODE region, ten to thirty fewer tagSNPs were selected by Snagger than Tagger (Table 1a.). TagSNPs chosen by Snagger had comparable, if not higher design scores than those selected by Tagger (Table 1b.). Also, tagSNPs selected by Snagger provided comparable coverage of all SNPs of interest to those selected by Tagger (Table 1c.).

### Selection of tagSNPs outside a targeted region

To evaluate Snagger's ability to select tagSNPs outside of a targeted region, we looked across 76 gene regions. There were a total of 6282 common (MAF = 0.05) SNPs targeted for capture using CEPH and Han Chinese genotypes from HapMap Public Release 21a [21] and the Affymetrix GeneChip Human Mapping 500 K Array set [22].

Choosing tagSNPs only from within each region yielded 1702 tagSNPs with an average Illumina design score of 0.902 (possible design scores were 0 through 1, or 1.1; a score of 1.1 is indicative of a successfully designed SNP assay). In either CEPH or Han Chinese populations, 75 SNPs were untaggable because they were within 60 base pairs of another tagSNP or had a design score equal to zero and could not be captured by any other tagSNP. In CEPH and Han Chinese populations there were 56 and 36 untaggable SNPs, respectively.

When we allowed tagSNPs to be picked from outside the region there were 1731 tagSNPs selected with an average design score of 0.917. In either CEPH or Han Chinese populations, there were 61 untaggable SNPs, and in CEPH and Han Chinese populations there were 45 and 28 untaggable SNPs, respectively.

## Discussion

The development and implementation of tagging SNP selection methodologies have received significant attention in recent years. Our program, Snagger, improves upon other tagSNP picking software by combining preferential tagSNP picking and the ability to select tagSNPs across multiple ethnic populations into one software package. With features not available in other software, including surrogate tagSNP picking to offset the risk of failed assays and the ability to pick better tagSNPs from outside a targeted region, Snagger improves coverage of genomic variation. Like Tagger, Snagger adds flexibility by allowing the user to force-include or force-exclude user-defined SNPs. The software is built on the basis of Haploview's framework, making it both familiar and graphically appealing to the user, and includes Haploview's LD plot and haplotype display, which allows the user to visually investigate patterns of variation.

Our program, Snagger, has some similarities to the algorithm used by Tagger. Both programs create a bin (BinA in Snagger) for each SNP that contains the set of SNPs in high LD (e.g., *r*^2 ^≥ 0.8). Snagger distinguishes itself from Tagger by including a second step. To preferentially select SNPs with certain characteristics, Snagger creates a set of bins (BinB) with SNPs that are in LD with every other SNP in the bin. Calculated from a number of user-specified parameters, a SNP Score is assigned to each SNP. The SNP in each BinB with the highest SNP Score becomes the potential set of tags to pick from. Thus, a SNP Score that can be flexibly weighted allows the user to influence the characteristics of chosen tagSNPs. The second set of bins maximizes coverage while minimizing the number of tagSNPs selected. From our evaluation, we show that Snagger on average selects fewer tagSNPs than the web server Tagger when preferentially selecting tagSNPs on design score.

Snagger offers the user the ability to preferentially pick SNPs that are located within a coding region or other genomic location. SNPs that either change an amino acid residue (known as non-synonymous SNPs) or are located in a 5' or 3' untranslated region are suspected to have a greater likelihood of having a biological effect [23,24]. The ability to prioritize these SNP offers added flexibility and many candidate-gene association studies of complex disease have included all known functional SNPs into their selection strategies. To our knowledge, no other available software package includes this feature.

Another key feature of Snagger is the ability to weigh by probability of successful genotyping on specific high-throughput platforms. Genotyping failures can reduce effective genomic coverage, especially when tagSNPs acting as a proxy for many SNPs fail. Snagger addresses this by preferentially choosing tagSNPs with high probabilities of success, while maintaining efficiency in the number of tagSNPs selected. Though it may be necessary for the software to pick tagSNPs with lower probabilities of success in order to capture every SNP, the user can enforce a minimum design score for all tagSNPs. For reference, 18% of HapMap SNPs in the ten HapMap ENCODE regions have a probability of success below 0.776, which corresponds to an Illumina design score of 0.4, the default minimum. In addition, Snagger can select surrogate tagSNPs that will backup low-scoring tagSNPs that act as a proxy for several SNPs. Furthermore, since some genotyping platforms (e.g., Illumina) require that all tagSNPs being genotyped have a minimum base pair distance, Snagger can enforce a minimum distance between tags, which further reduces the chance of genotyping failure. We compared our program to the web server Tagger and show that the tagSNPs chosen by Snagger had comparable, if not higher design scores than those selected by Tagger.

Snagger's ability to select tagSNPs across multiple populations in a user-friendly manner is advantageous for studies involving multi-ethnic cohorts and admixed populations. Other software programs have focused on the most efficient way to select tags including TAGster [19], but do not include other features available in Snagger. Furthermore, we are currently extending the selection algorithm to incorporate haplotype information in addition to pairwise LD.

## Conclusion

We developed a software application, Snagger, to select an efficient set of tagSNPs that captures the most genetic information and can reliably be genotyped. It is freely available and we include the executable (see Additional File 1), source code (see Additional File 2), user guide (see Additional File 3), sample SNP information (see Additional File 4), and sample HapMap data (see Additional File 5). It performs better than the web server Tagger by choosing fewer tagSNPs when weighting on design score, and performs equally as well in selecting tagSNPs that provide comparable coverage of genomic regions that can be genotyped successfully. In addition, our software program allows the user to conveniently select tagSNPs across multiple populations as well as from outside gene regions of interest, and to include surrogate tagSNPs as another way to offset the risk of failed assays. Moreover, Snagger allows the user to incorporate the probability of genotyping success in the SNP selection process and to give greater priority to, and subsequently choose, particular types of SNPs by functionality, location and MAF. These capabilities significantly improve upon current available tagSNP software packages.

## Availability and requirements

Project Name: Snagger

Project home page and availability: 

Operating system(s): Platform independent

Programming language: Java

Other requirements: Java Runtime Environment 1.4.2_12 or higher

License: MIT License

Any restrictions to use by non-academics: None

## Authors' contributions

CKE carried out the software engineering effort and performance analysis and participated in the drafting of the manuscript. WHL participated in the development of an alpha version of the program and the drafting of the manuscript. DL participated in the development of an alpha version of the program. DJV and DVC supervised the development of the program and the drafting of the manuscript. All authors read and approved the final manuscript.

## Appendix

### Tagging algorithm summary (see Figure 3 for overview)

Input:

• A set of SNPs S = {s_1_, s_2_,..., s_m_} within a contiguous genomic region.

• A table containing *r*^2 ^values for each pair of SNPs in S having a physical distance less than a user-specified threshold, such that the pairwise *r*^2 ^value of two SNPs s_i _and s_j _is defined as: *r*^2^(s_i_, s_j_).

• A set of SNPs I = {i_1_, i_2_,..., i_n_} to force-include as chosen tagSNPs, where I ⊆ S.

• A set of SNPs E = {e_1_, e_2_,..., e_o_} to force-exclude from being chosen as tags, where E ⊆ S.

• A user-specified *r*^2 ^minimum threshold defined as: *r*^2^_min_. All tagSNP-SNP pairs must have a pairwise *r*^2 ^value that meets or exceeds this threshold.

• A SNP Score function *SS *based on SNP design scores, other annotations, and user-defined weights.

Output:

• A set of tagSNPs T = {t_1_, t_2_,..., t_r_} such that T ⊆ S and each t ∈ T tags a subset of SNPs in S.

• A set of "untaggable" SNPs U = {u_1_, u_2_,..., u_s_} such that U ⊆ S and no tag SNP in T tags any SNP in U.

Algorithm:

1) Let C = {c_1_, c_2_,..., c_p_} be the remaining set of SNPs to capture, such that C initially contains the SNPs in S that are located within the region of interest.

*C *⊆ *S*.

2) Add all force-included SNPs to the final list of tagSNPs.

*For each i*_*i *_∈ *I, add i*_*i *_*to T*.

3) Remove all SNPs from the set of SNPs that still need to be captured those SNPs that are tagged by the set of force-included tagSNPs.

*For all possible pairs of t*_*i *_∈ *I and c*_*j *_∈ *C, if r*^2^*(t*_*i*_, *c*_*j*_*) *≥ *r*^2^_*min*_, *remove c*_*j*_* from C*.

4) Determine the remaining set of SNPs that can possibly be tagSNPs.

*Let P = {p_1_, p_2_,...,p_*q*_} be the set of potential tagSNPs such that P = S - E - I*.

5) Determine the set of SNPs for which each potential tagSNP can act as a proxy based on their pairwise *r*^2 ^values.

*For each p*_*i *_∈ *P, create a BinA such that p.BinA ⊆ S. For each c*_*j *_∈ *C where r*^2^*(p*_*i*_, *c*_*j*_*) = r*^2^_*min*_, *add c*_*j *_*to p.BinA*.

6) For each potential tagSNP, find a set of potential tagSNPs that can act as proxies for it and every other potential tag in the set.

*For each p*_*i *_∈ *P, create a BinB such that p. BinB ⊆ P and p*_*i *_∈ *p. BinB, where all possible pairs of b*_*j *_∈ *p. BinB and c*_*k *_∈ *p. BinB, r*^2^(*b*_*j*_, *c*_*k*_) ≥ *r*^2^_*min*_.

7) For each potential tag, determine its best proxy according to the user-defined scoring function *SS *(e.g., highest probability of genotyping success) and add it to a temporary set.

*Let Q = {}. For each p*_*i *_∈ *P, let p*_*i*-*best *_*be the SNP with the highest SNP Score, SS, in p*_*i*_.*BinB. Add p*_*i-best *_*to Q*.

8) From the temporary set of best proxies, choose the SNP that tags the most number of SNPs and add it to the final set of tagSNPs.

*Let t be the SNP in Q with the largest BinA. Add t to the set of tagSNPs T*.

9) Create a set of SNPs that will be removed from the list of potential tags.

*Let R = {}. Add t to R*.

10) Remove all the newly tagged SNPs from every potential tagSNP's BinA. If a potential tagSNP's BinA becomes empty, it can no longer be a tag and should be removed.

*For each p*_*i *_∈ *P, p. BinA = p. BinA - t. BinA. If p*_*i*_*.BinA = {}, add p*_*i *_*to R*.

11) Remove all newly tagged SNPs from the set of SNPs that are left to be captured.

*Let C = C - t. BinA*.

12) Remove all the potential tags that were marked for removal from every potential tagSNP's BinB.

*For each p*_*i *_∈ *P, let p*_*i*_*.BinB = p*_*i*_*.BinB - R*.

13) Remove all the potential tags that were marked for removal from the set of potential tagSNPs.

*Let P = P - R*.

14) If there are no more SNPs to capture or there are no more potential tagSNPs to choose from (i.e., if C = {} or P = {}), the tagSNP picking is done. Otherwise, choose the next tagSNP by repeating from Step 7.

15) Mark any SNPs that still need to be captured as untaggable.

*Let U = C*.

## Supplementary Material

Additional file 1 **Haploview with Snagger as an executable JAR file**. The file is contained inside a .zip file, and must be extracted before it can be accessed. This file contains the program Snagger as an extension to Haploview version 3.3. It can be executed on any operating system with the Sun Java Runtime Environment version 1.4.2_12 or higher installed [25]. It does not work with gcj or kjc. To start it from a command line, type: 'java -jar 1471-2105-9-174-S1.jar'. Click here for file

Additional file 2 **Archive of source code for Haploview with Snagger**. This file contains an archive of the source code for Haploview with Snagger. It is compressed using the GNU Tar and GNU zip (gzip) archive utilities inside a .zip file, in that order. To extract the archive with Windows, use WinZip [26]. For Unix-based operating systems, use the command: 'tar xvzf HaploviewWithSnagger_v1.07.tar.gz'. For other operating systems, first uncompress with GNU zip [27] then untar with GNU Tar [28]. For compilation instructions, see the README file contained in the archive. Click here for file

Additional file 3 **Haploview with Snagger User Guide**. This file contains a user guide in PDF format with instructions on how to use Snagger. General Haploview instructions not relating to Snagger are available from the Help menu of the program. Click here for file

Additional file 4 **Sample score file**. This file is an example score file that will work with Snagger. It should be input in the software's window titled: "Load Score File and HapMap Data". Click here for file

Additional file 5 **Archived folder of sample HapMap data**. This file contains an archived folder of sample HapMap data that corresponds with the SNPs in the sample score file. It is in GNU Tar format and should be uncompressed to a local hard disk before using. The resulting uncompressed folder should be specified in the software's window titled: "Load Score File and HapMap Data". To extract on Windows, use WinZip [26]. For Unix-based operating systems, use the command: 'tar xvf 1471-2105-9-174-S5.tar'. For other operating systems, untar the archive with GNU Tar [28]. Click here for file
