# Supplementary material for: Snagger: A user-friendly program for incorporating additional information for tagSNP selection
Source: BMC Bioinformatics. 2008 Mar 27;9:174. doi: 10.1186/1471-2105-9-174 (PMC2375134; doi:10.1186/1471-2105-9-174)
Supplement: Additional file 3 — Haploview with Snagger User Guide. This file contains a user guide in PDF format with instructions on how to use Snagger. General Haploview instructions not relating to Snagger are available from the Help menu of the program. [file 1471-2105-9-174-S3.pdf]

Haploview with Snagger 1.07 User Guide

2008-03-12

1) OVERVIEW 2

2) INSTALLATION AND SETUP 2

A) WINDOWS, MAC OSX, LINUX, ETC. 2

3) SYSTEM REQUIREMENTS 2

4) USING SNAGGER 3

A) LOADING A DATASET 3

i) Score File 3

Required columns: 4

Optional columns: 4

ii) HapMap files 4

iii) Genomic Region 5

iv) Populations 5

B) DATA QUALITY CHECKS 6

C) RUNNING SNAGGER 7

i) r^2 Threshold 8

ii) Minimum Distance between Tags 8

iii) Picking TagSNPs From Outside the Region 8

iv) Surrogates 9

v) Minimum Design Score 10

vi) Advanced Options (Weighting on Additional SNP Information) 11

D) RUNNING MULTIPLE POPULATIONS SNAGGER 13

E) SNAGGER RESULTS 14

5) COMMAND LINE OPTIONS 15

A) GENERAL OPTIONS 15

B) INPUT OPTIONS 16

C) DATA CHECK OPTIONS 16

D) BLOCK OUTPUT OPTIONS 17

E) OTHER OUTPUT OPTIONS 18

F) TAGGER OUTPUT OPTIONS 18

G) SNAGGER OUTPUT OPTIONS 19

1) Overview

We have developed a user-friendly and efficient software program, Snagger, as an extension to the existing open-source software, Haploview, which uses pairwise r^2 linkage disequilibrium between single nucleotide polymorphisms (SNPs) to select tagSNPs. Snagger distinguishes itself from existing SNP selection algorithms, including Tagger, by providing user options that allow for: (1) prioritization of tagSNPs based on certain characteristics, including platform-specific design scores, functionality (i.e. coding status), and chromosomal position, (2) efficient selection of SNPs across multiple populations, (3) selection of tagSNPs outside defined genomic regions to improve coverage and genotyping success, and (4) additional picking of surrogate tagSNPs that can act as backups for tagSNPs having a significant failure risk. Using HapMap genotype data from ten ENCODE regions and design scores for the Illumina platform, we show similar coverage and design score distribution and fewer total tagSNPs selected by Snagger compared to the web server Tagger.

Snagger improves upon current available tagSNP software packages by providing a means for researchers to select tagSNPs that reliably capture genetic variation across multiple populations while accounting for significant genotyping failure risk and prioritizing on SNP-specific characteristics.

Haploview with Snagger has been developed and is maintained at the Genomics Center at the University of Southern California. For issues concerning installation or usage, please refer to the project website: http://sourceforge.net/projects/snagger.

2) Installation and Setup

a) Windows, Mac OSX, Linux, etc.

Haploview with Snagger is developed in Java, making it a platform-independent program. It can be run by typing `java -jar HaploviewWithSnagger.jar` on the command line. If your operating system is configured correctly, you should be able to start the program by double-clicking on the jar file.

3) System Requirements

It is recommended that Haploview with Snagger be run on a machine with at least 128MB of memory.

Haploview with Snagger requires the Sun Java Runtime Environment 1.4.2_12 or later. It does not work with gcj or kjc. You can download the most recent Java release at http://java.sun.com/javase/downloads/index.jsp.

4) Using Snagger

a) Loading a Dataset

When starting Haploview with Snagger, a window titled, **Load Score File and HapMap Data** appears (see Figure 1). Here you must specify certain options in order to load data into Haploview with Snagger. See below for descriptions of the fields. Click **OK** to load the data into Haploview with Snagger.

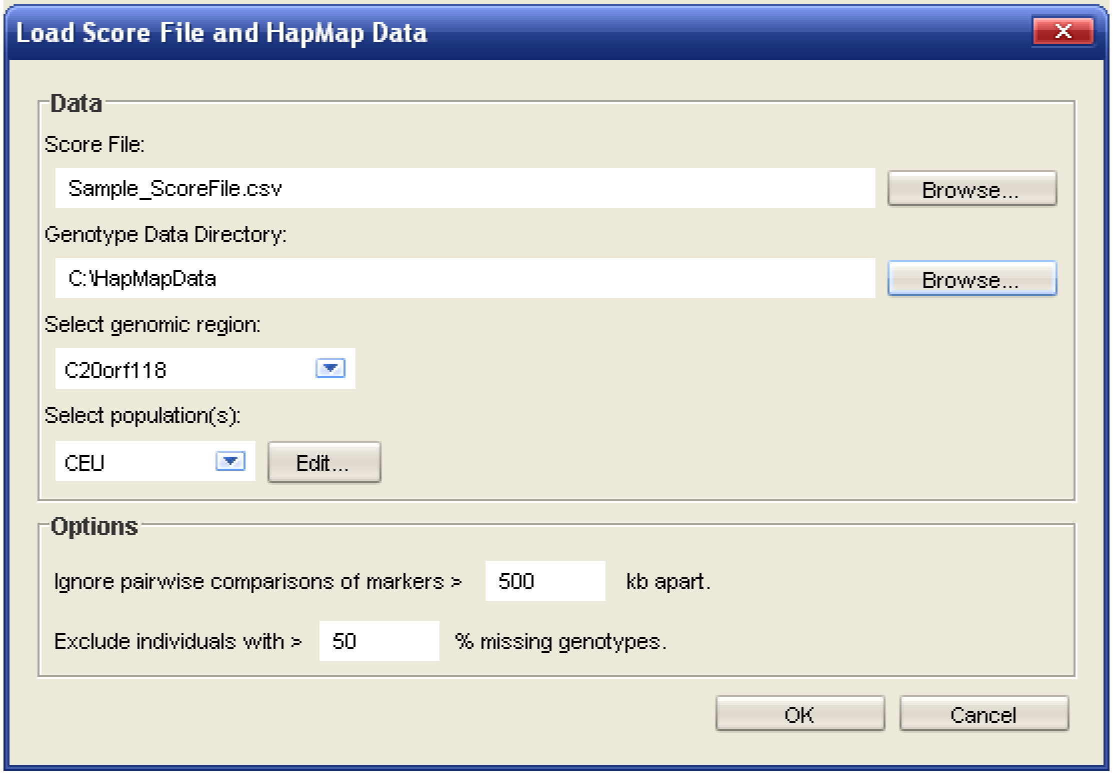

Figure 1 – Load Score File and HapMap Data window

i) Score File

You must specify the path to a **Score File** (comma delimited) containing the following columns:

Required columns:

Column Name	Description
Snp_Name	SNP identifiers. Each name in the file must be unique.
Chr	The chromosome which the SNP is mapped to (e.g. '1', 'X')
Coordinate	The chromosomal position which the SNP is mapped to.
Customer_Annotation	The name of the gene region this SNP is associated with. All SNPs within the same region must share the same chromosome.
SNP_Score	A design score ranging from 0-1.0 or 1.1

Optional columns:

Column Name	Description
Force_Include	Either 0 or 1. A value of 1 indicates this SNP should be force-included as a tag regardless of any filters or missing genotype data. The Force_Capture column should have a value of 1 if this column is 1. Values of 0 are treated as normal.
Force_Capture	Either 0 or 1. A value of 1 indicates this SNP should be force-captured. That is, it should be tagged by another SNP or chosen as a tag regardless of any filters or missing genotype data. Values of 0 are treated as normal.
Potential_Tag_No_Capture	Either 0 or 1. A value of 1 indicates this SNP should not be captured by Snagger, but can be a potential tag for other SNPs that should be captured. These SNPs typically reside outside of the region of interest.
Location	For use with advanced Snagger options. Values can be: '3UTR', '5UTR', 'UTR', 'coding', 'flanking_3UTR', 'flanking_5UTR', 'intron', or '-1' (i.e. unknown).
Coding_Status	For use with advanced Snagger options. Values can be: 'SYNON', 'NONSYN', or '-1' (i.e. unknown).

ii) HapMap files

You must also specify the directory containing the set of genotype data files. Each genotype file must be in HapMap format (see the Haploview Help file) and be present in the **Genotype Data Directory**, specified in the **'Load Score File and HapMap Data'** dialog window. In order for Haploview with Snagger to recognize the file, it must have the following format:

hapmap_{REGION NAME}_{POPULATION ID}.txt

Examples:

hapmap_VDR_CEU.txt

hapmap_ESR1_CHB+JPT.txt

hapmap_H19_YRI.txt

hapmap_PTGS1_CEU+CHB+JPT+YRI.txt

Note that when combining HapMap populations into a group, the population should be the concatenation of the population IDs with the plus (‘+’) character as a separator. The population IDs must be in alphabetical order.

Important: You must ensure that the HapMap files contain the exact SNP identifiers that are present in the Score file in order for all the data to be loaded properly.

iii) Genomic Region

Each time a dataset is loaded, you must specify a genomic region in the drop-down control. Only the regions listed in the Score File will be displayed. You can load a new region by returning to the “Load Score File and HapMap Data” window via the “File” menu (see Figure 1).

iv) Populations

The HapMap population you wish to load can be chosen with the drop-down control. The four HapMap populations are listed by default: CEU, CHB, JPT and YRI. If you want to combine populations or tag multiple populations in succession, you can click the “Edit” button next to the drop-down (see Figure 2).

In order to edit a population group or snagging order, you should click the “New” button under the “Snagging Macros” list. Assign the macro a name and highlight it to start editing (default populations cannot be edited).

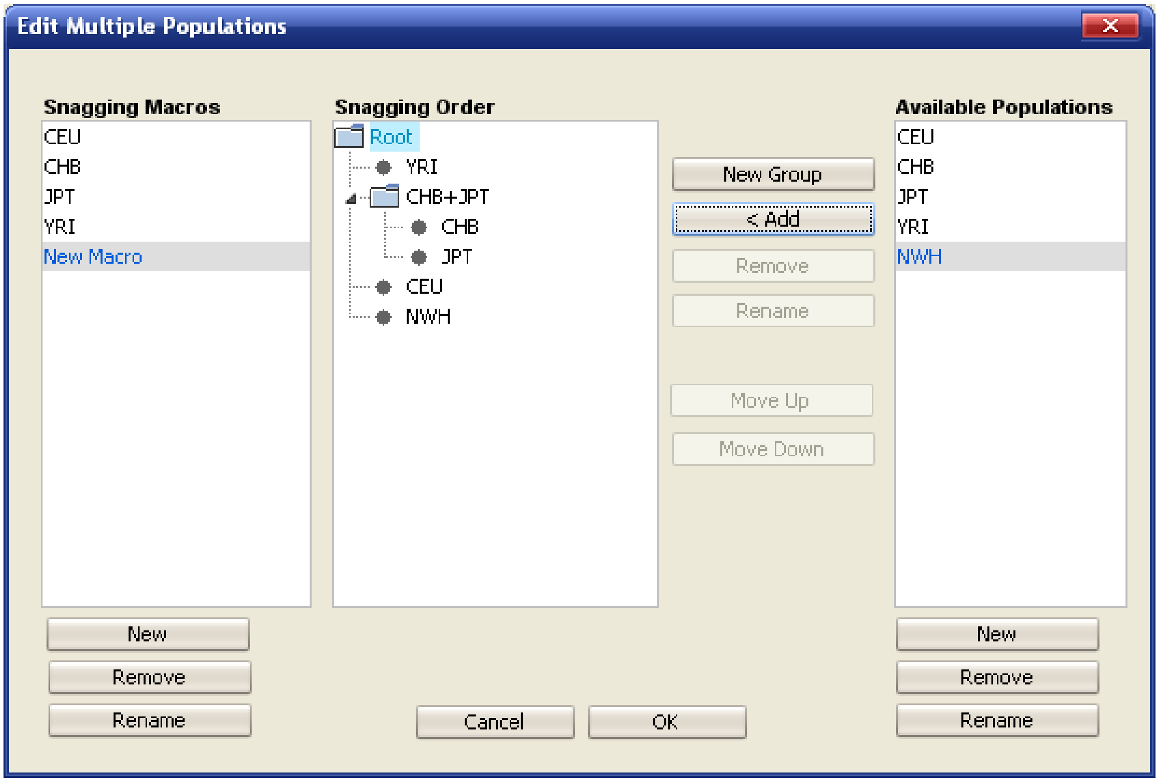

Figure 2 – Edit Multiple Populations window.

The "Snagging Order" defaults to one folder named "Root". You can add populations in the order for which you would like to pick tags by clicking the "Add" button. Click the "Move Up" or "Move Down" button to alter the order of tag picking.

Population groups can be added to the snagging macro by clicking the "New Group" button. Any populations you add while having the group highlighted will be combined into one population. You can add custom populations by clicking the "New" button underneath the "Available Populations" list.

See 4)d) Running Multiple Populations Snagger

b) Data Quality Checks

Once you have loaded data for a particular genomic region, you can filter which SNPs you want to capture. See the Haploview help section (contained within the program) entitled "Data Quality Checks".

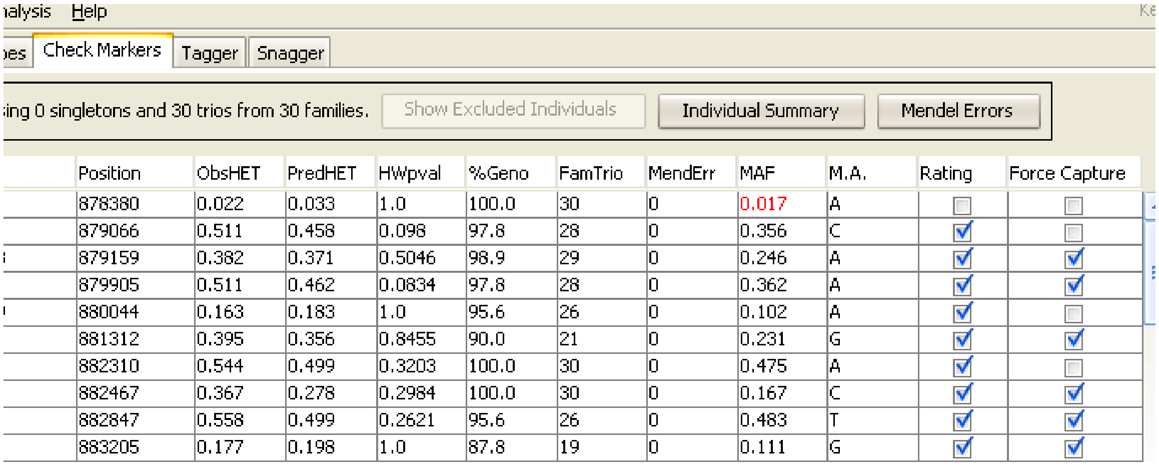

Position	ObsHET	PredHET	HWpval	%Geno	FamTrio	MendErr	MAF	M.A.	Rating	Force Capture
878380	0.022	0.033	1.0	100.0	30	0	0.017	A	☐	☐
879066	0.511	0.458	0.098	97.8	28	0	0.356	C	☑	☐
879159	0.382	0.371	0.5046	98.9	29	0	0.246	A	☑	☑
879905	0.511	0.462	0.0834	97.8	28	0	0.362	A	☑	☑
880044	0.163	0.183	1.0	95.6	26	0	0.102	A	☑	☐
881312	0.395	0.356	0.8455	90.0	21	0	0.231	G	☑	☑
882310	0.544	0.499	0.3203	100.0	30	0	0.475	A	☑	☐
882467	0.367	0.278	0.2984	100.0	30	0	0.167	C	☑	☑
882847	0.558	0.499	0.2621	95.6	26	0	0.483	T	☑	☑
883205	0.177	0.198	1.0	87.8	19	0	0.111	G	☑	☑

Figure 3 – Check Markers tab

An additional column on the **Check Markers** tab is included with Snagger named **Force Capture** (see Figure 3). If a checkbox for a marker is checked, it will be captured without regard to any filters you specify on this tab. If your Score File has a ‘Force_Capture’ column, the checkboxes in this column will be automatically initialized.

When loading data from the USC Genomics Core database, all force-captured markers will be loaded into Haploview with Snagger, even those not existing in the database.

c) Running Snagger

The Snagger Configuration Tab (see Figure 4) allows you to specify the options you would like to use to run Snagger. A table including all the SNPs which pass the criteria you set on the Check Markers tab is displayed. You can choose to force include or force exclude SNPs as tagSNPs here by selecting the appropriate checkboxes next to the SNPs you wish to force include or force exclude. The Force Include column here will be automatically initialized to match your Score File’s ‘Force_Include’ column (if it exists). To run Snagger, click the **Run Snagger** button. The results will be displayed on the Snagger Results tab (see 4)e) Snagger Results).

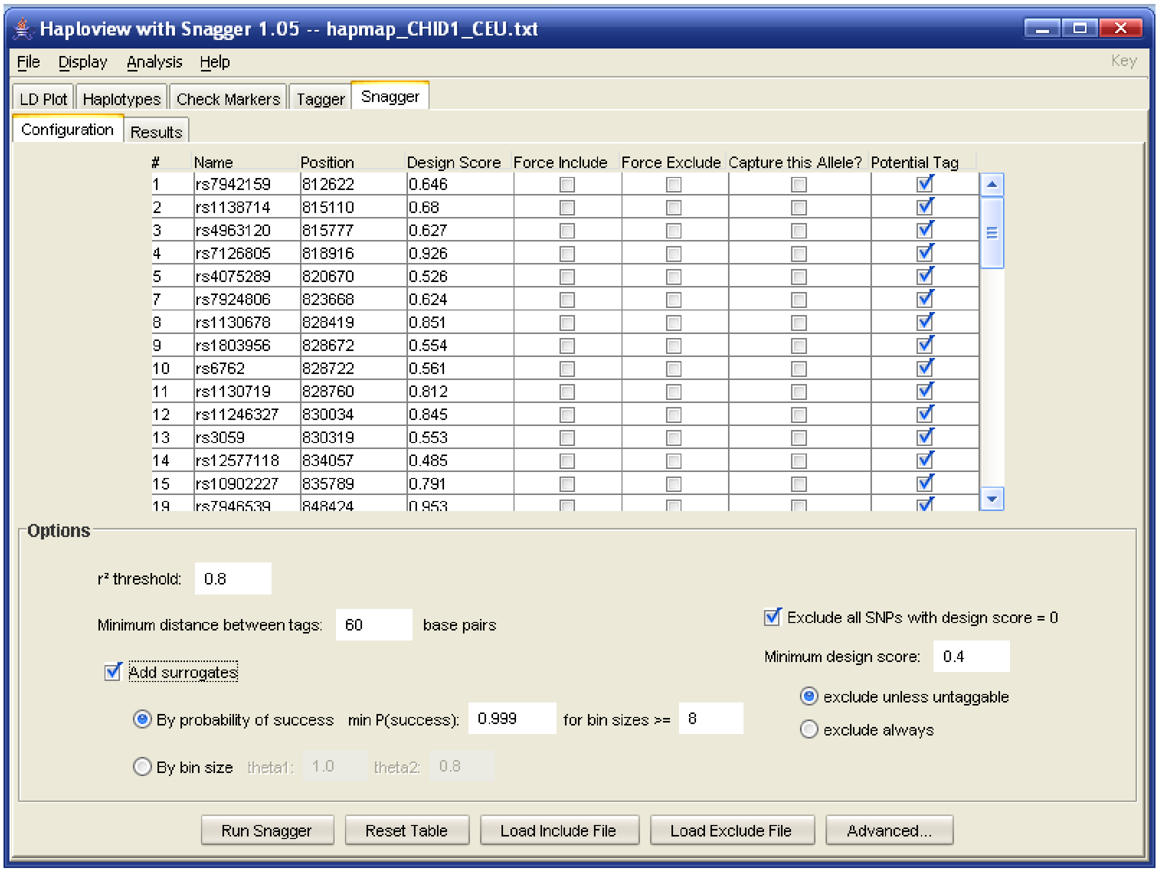

Figure 4 – Snagger Configuration tab

i) r² Threshold

You can specify the tagging r² threshold on this tab. Snagger will attempt to tag each marker in the filtered list with a pair-wise r² value greater than or equal to the threshold you specify.

ii) Minimum Distance between Tags

Snagger offers the ability to enforce a minimum distance (in base pairs) between tagSNPs. A distance of 60 base pairs is required for the Illumina genotyping platform and is the default setting for this option.

iii) Picking TagSNPs From Outside the Region

Snagger can pick tagSNPs from outside of the target genomic region. This will increase the potential set of tagSNPs to pick from, increase the chances of finding high-quality tagSNPs and reduce the chances that SNPs within the region will be

"untaggable". To do this, you must include SNPs from outside of the region in both your HapMap data file and Score File. To specify which SNPs are outside of the region, and should be considered potential tagSNPs, you must have a column in the Score File named, 'Potential_Tag_No_Capture'. Any SNPs outside of the region that should not be captured, but can be picked as tagSNPs should have a value of 1 for this column. All other SNPs should have a value of 0. See 1)a)i) Score File. These values are reflected in the **'Capture this Allele?'** and **'Potential Tag'** columns of the Snagger Configuration Tab. See Figure 4 – Snagger Configuration tab.

iv) Surrogates

You can choose the option to add surrogate tagSNPs for tagSNPs that are tagging a large number of markers. There are two methods for surrogate picking:

(1) By probability of success – All markers are assigned a calculated probability of success (CPS) based on the design score of all the tagSNPs that are tagging them. For instance, a marker that is being tagged by two tagSNPs with genotyping probability of successes (GPS) of 0.99 and 0.95 would have a resulting CPS = 1 – (1 – 0.99) x (1 – 0.95) = 0.9995.

Thus, the more tagSNPs a marker has, the higher its CPS will be. The GPS function is based on experimental data obtained from genotyping experiments performed on the Illumina Bead Lab platform at the USC Genomics Center. The function is:

$$\hat{\pi} = \exp(\hat{\alpha} + \hat{\beta}X) / (1 + \exp(\hat{\alpha} + \hat{\beta}X))$$

$$\hat{\alpha} = -0.27055$$

$$\hat{\beta} = 3.776686$$

Where, $\hat{\pi}$ = GPS (genotyping probability of success), and X = design score.

Choosing this option would require a minimum CPS (specified in the **min P(success)** textbox) for each marker in the bin of a tagSNP having a bin size greater than or equal to the value specified in the **for bin sizes >=** textbox. The bin size is equal to the number of markers in linkage disequilibrium (based on the r^2 threshold) with the tagSNP and having a CPS less than the cutoff. The alpha and beta values in the function above are Illumina-specific. These values can be changed in the Snagger Advanced options. See 4)c)vi)(1) Probability of Success – The default method for picking tagSNPs. The genotyping probability of success is based on the design score. Identified as $Pr.S_m$ in Figure 7.

(2) By bin size – The second option for adding surrogates uses only the bin size of the tag to determine how many surrogates to add. The bin size has a slightly different definition for this option, and is just the number of markers in linkage disequilibrium (based on the r^2 threshold) with the tagSNP.

You can specify values for **theta1** and **theta2** such that:

S: number of surrogates needed
B: bin size

S = log (theta1*B^theta2) - 1

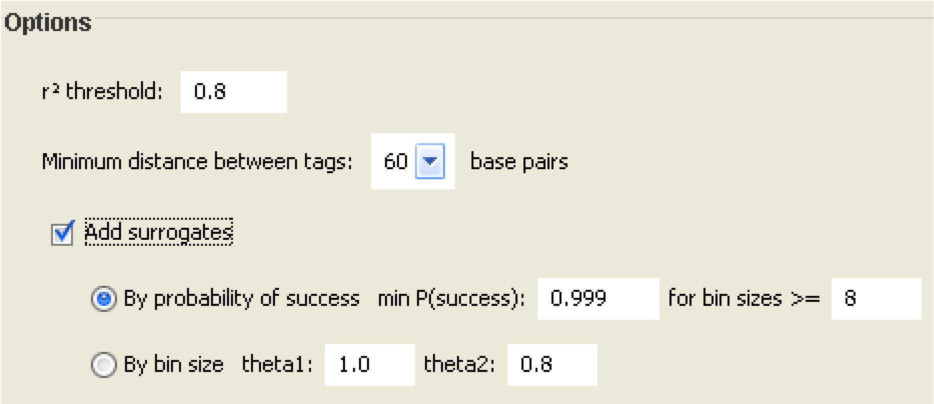

Figure 5 – Snagger Options (r^2 threshold, min tag distance, surrogates)

v) Minimum Design Score

You can prevent tagSNPs from being chosen that do not meet a minimum design score threshold by specifying a value from 0 to 1.1. By default, all SNPs with a design score less than 0.4 will be excluded, unless they become untaggable, in which case they must be chosen as tags.

You can choose to always exclude SNPs below the design score cutoff by selecting the **exclude always** option.

In addition, you can choose to exclude all SNPs with a design score of zero.

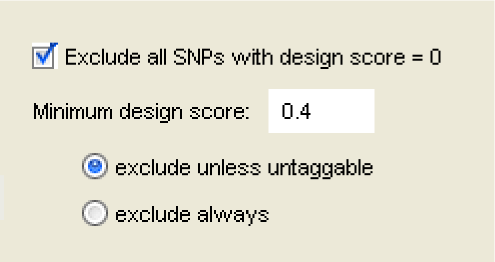

Figure 6 – Snagger Options (Minimum design score)

vi) Advanced Options (Weighting on Additional SNP Information)

By default, Snagger will preferentially pick tagSNPs with a high design score (i.e. high probability of genotyping success). If you wish to preferentially pick tagSNPs using other criteria, you can do so by clicking on the **Advanced…**button (see Figure 8). Here you can give a numerical weighting to four different categories:

$$S.u_m = w.\Pr.S * \Pr.S_m + w.MAF * MAF.S_m + w.Type * Type.S_m + w.Loc * Loc.S_m$$

$$\Pr.S_m = \frac{\exp(-0.27 + 3.78 * DesignScore)}{1 + \exp(-0.27 + 3.78 * DesignScore)}$$

$$MAF.S_m = \frac{\sum_{p=1}^{P} MAF.S_{mp}}{P}$$

$$MAF.S_{mp} = 1 - \frac{abs(idealMAF - observedMAF_{mp})}{0.5}$$

$$idealMAF = \text{user defined}$$

$$Type.S_m = \begin{cases} Type.S_{-1} & \text{user defined between 0 and 1} \\ Type.S_{NONSYN} & \text{user defined between 0 and 1} \\ Type.S_{SYNON} & \text{user defined between 0 and 1} \end{cases}$$

$$Loc.S_m = \begin{cases} Loc.S_{3UTR} & \text{user defined between 0 and 1} \\ Loc.S_{5UTR} & \text{user defined between 0 and 1} \\ Loc.S_{coding} & \text{user defined between 0 and 1} \\ Loc.S_{flanking\_3UTR} & \text{user defined between 0 and 1} \\ Loc.S_{flanking\_5UTR} & \text{user defined between 0 and 1} \\ Loc.S_{intron} & \text{user defined between 0 and 1} \end{cases}$$

Figure 7 – Advanced scoring functions

(1) **Probability of Success** – The default method for picking tagSNPs. The genotyping probability of success is based on the design score. Identified as $Pr.S_m$ in Figure 7. The alpha and beta coefficients are defaulted for the Illumina genotyping platform. These values can be changed in the Snagger Advanced Options window.

(2) **MAF (minor allele frequency) –** You can give a weight to the average minor allele frequency of the marker across all populations such that the closer it is to a user defined "**Ideal MAF**", the higher its score will be. Identified as $MAF.S_m$ in Figure 7.

(3) **Amino Acid Change –** This category requires that the Score File contains the column, "Coding_Status", with values equal to 'SYNON' (i.e. synonymous), 'NONSYN' (i.e. non-synonymous) , or '-99' (i.e. unknown). You can assign different scores (between 0 and 1) to each of the different amino acid change categories. Identified as $Type.S_m$ in Figure 7.

(4) **Locus –** This category requires that the Score File contains the column, "Location", with values equal to '3UTR', '5UTR', 'UTR', 'coding', 'flanking_3UTR', 'flanking_5UTR', 'intron', or '-99' (i.e. unknown). You can assign different scores (between 0 and 1) to each of the different location categories. Identified as $Loc.S_m$ in Figure 7.

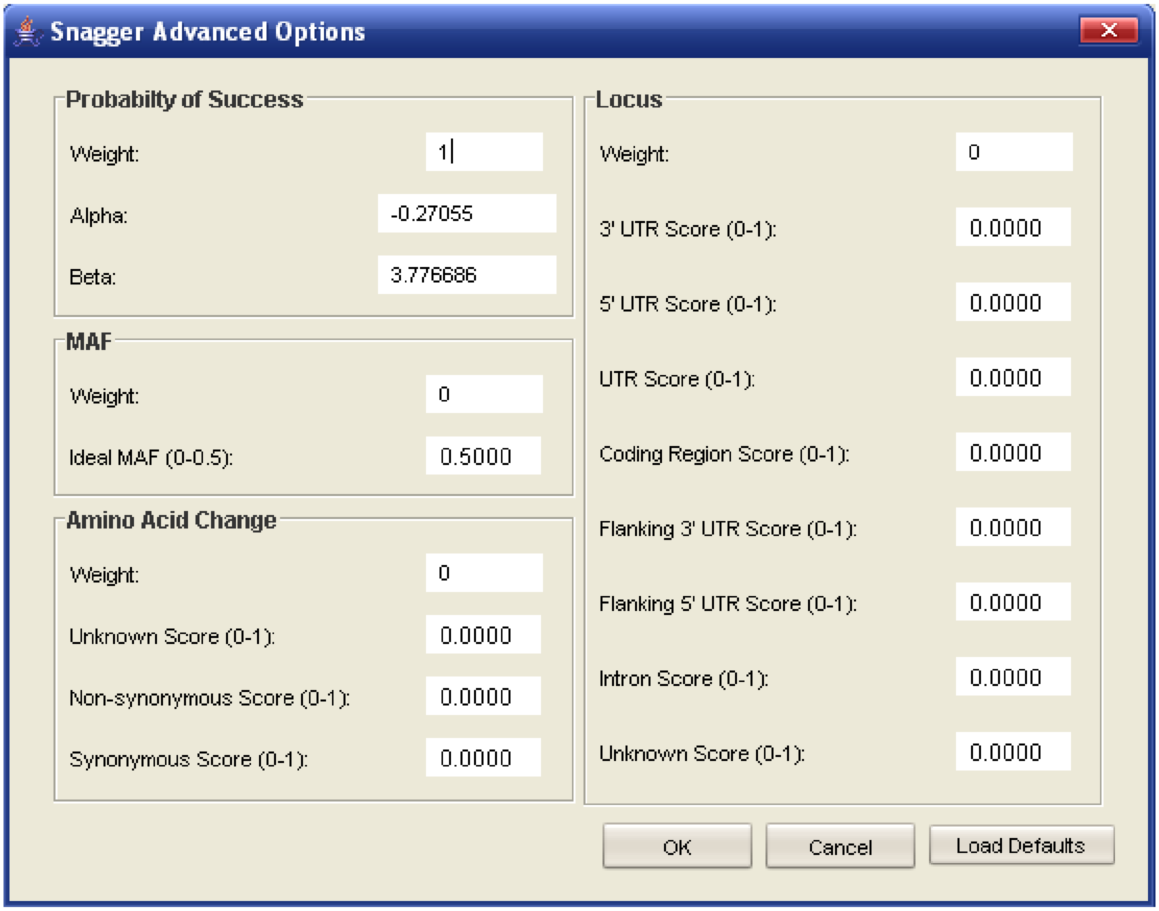

Figure 8 – Snagger Advanced Options window

d) Running Multiple Populations Snagger

When loading more than one population into Haploview with Snagger, you will see a new set of tabs at the top of the window. The first will be the **Multiple Populations Snagger** tab containing **Configuration** and **Results** sub tabs. There will also be a tab for each population you've added (shown in the same order that they will be tagged). The only difference you will see on these population tabs is that they are missing Snagger configuration options. Instead, the options for each of the populations are combined into one set of options on the **Multiple Populations Snagger Configuration** tab. Any options you specify here will be used for each of the populations' Snagger configuration.

Multiple Populations Snagger works by picking tags for the first population and forcing them in as tags on subsequent populations. Additional tags are picked as required in the populations that follow.

To run Multiple Populations Snagger, specify the options you would like to use and click the **Run Snagger** button (see Figure 9). The results are displayed on the **Multiple Populations Snagger Results** tab (see Figure 10)

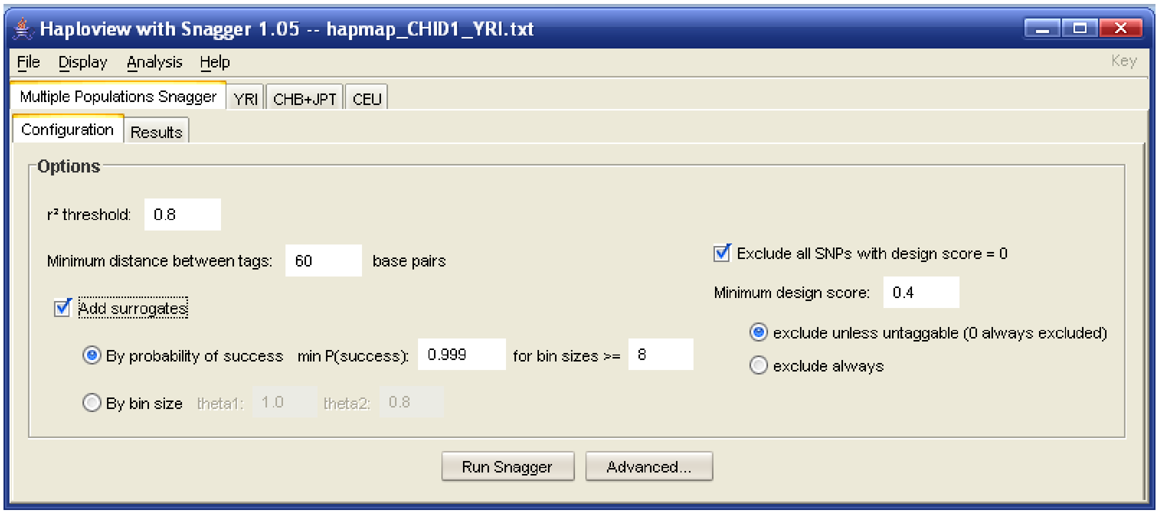

Figure 9 – Multiple Populations Snagger Configuration tab

e) Snagger Results

On this tab, you can see the results from Snagger or Multiple Populations Snagger.

The tree in the upper-left labeled **"Tags"**, shows all the tagSNPs as folders. Expanding a tagSNP folder reveals the markers that it tags.

Highlighting one or more tagSNP folders will change the Tests table contents to include the tag-marker pairs that are highlighted. The columns in the table include the tag name, marker name, pair-wise r^2 value, the tag's genotyping probability of success (GPS) and the marker's calculated probability of success (CPS) (see 4)c)iv)(1). If in Multiple Populations Snagger mode, each population pair-wise r^2 and P(s) columns will be displayed.

The list in the lower left contains all the markers that were tagged. Highlighting a marker will show what tagSNPs are tagging that marker in the **Tests** table.

If a marker is untaggable, it will be highlighted red in the **Tests** table and its tag will be labeled as "Untaggable". If there are multiple populations, only the r^2 columns for the populations for which the marker is untaggable will be highlighted red and have a value of 0.0 for r^2.

The tag list or table can be exported to a file by clicking the **Save Tags File…** or **Save Table File…** buttons respectively. To export all relavent files (including Check Markers, Haplotype Text, Haplotype Image, LD Plot, LD Table, Snagger Results Table, Snagger Summary and Tag List), you should click the **Save All...** button and choose a directory.

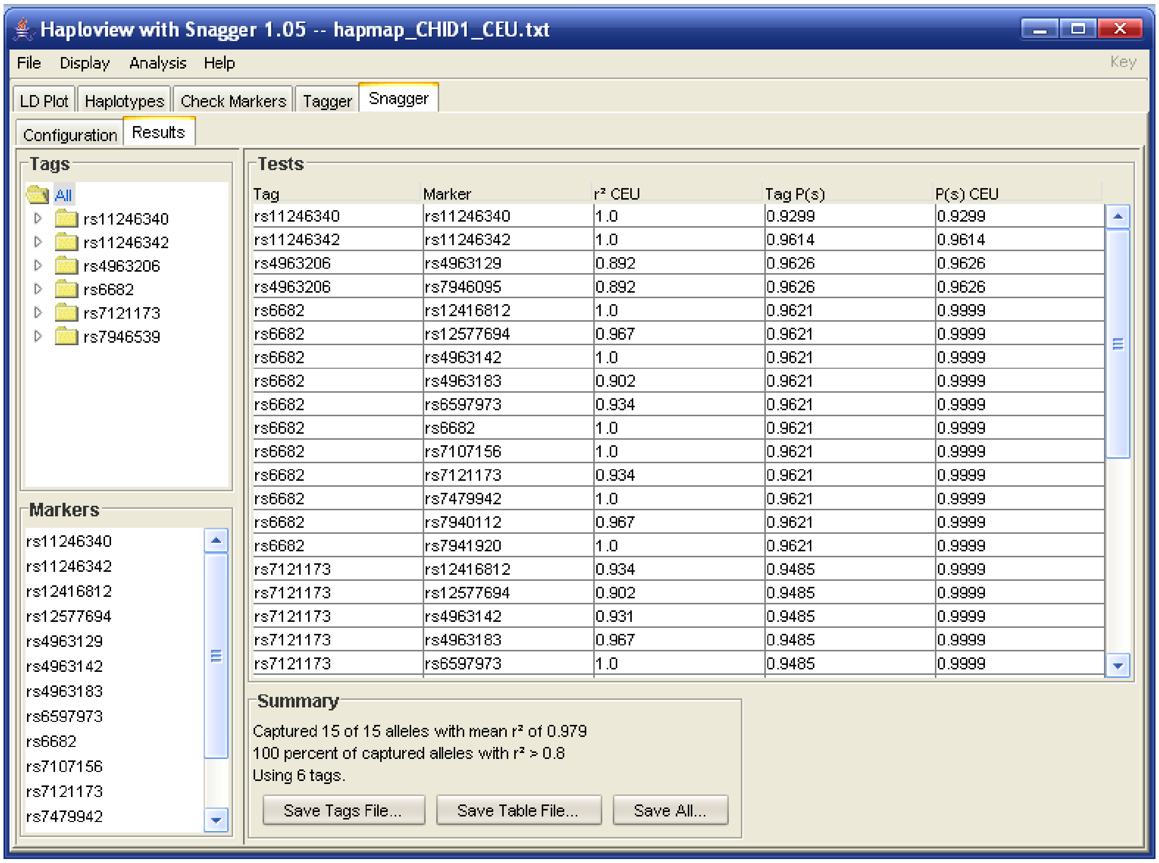

Figure 10 – Snagger Results tab

5) Command Line Options

Haploview with Snagger can be run from the command line without the display in order to process multiple gene regions and multiple configurations without user interaction. In order to run Haploview with Snagger without the display, add the "-nogui" flag. Haploview with Snagger can be run from the command line using:

java –jar HaploviewWithSnagger.jar

a) *General Options*

-h, -help

Print help information.

-n, -nogui

Command line mode—does not launch display.

-q, -quiet

Quiet mode—minimizes output to command line.

-memory <memsize>

Allocate <memsize> megabytes of memory to the Haploview with Snagger process (default is 512MB).

b) Input Options

-hapmap <filename>

Specify a HapMap format input file. This option works in GUI mode. (See original Haploview Help file)

-info <filename>

Specify a marker information file. This option works in GUI mode. (See original Haploview Help file)

-blocks <filename>

Specify a block definition file. This will automatically use this block definition for haplotype output. (See original Haploview Help file)

-track <filename>

Specify an analysis track file. (See original Haploview Help file)

-chromosome <1-22,X>

Specify which chromosome these data come from. This is especially critical when analyzing data from the X chromosome.

c) Data Check Options

-skipcheck

Skip all the genotype data quality checks and uses all markers for all analyses.

-minMAF <threshold>

Exclude all markers with minor allele frequency below <threshold>, which must be between 0 and 0.5. Default of 0. This option works in GUI mode.

-maxMendel <integer>

Exclude markers with greater than <integer> Mendelian inheritance errors. Default of 1. This option works in GUI mode.

-minGeno <threshold>

Exclude markers with less than <threshold> fraction of nonzero genotypes. <threshold> must be between 0 and 1 with a default of 0.5. This option works in GUI mode.

-hwcutoff <threshold>

Exclude markers with a Hardy Weinberg p-value less than <threshold>, which ranges from 0 to 1 with a default of 0.001 This option works in GUI mode.

-maxDistance <distance>

Maximum intermarker distance for LD comparisons (in kilobases). Default is 500. This option works in GUI mode.

-missingCutoff <threshold>

Exclude individuals with more than <threshold> fraction missing data, where <threshold> is a value between 0 and 1 with a default of 0.5. This option works in GUI mode.

d) Block Output Options

-blockoutput <type>

Generate haplotypes and population frequencies for blocks of <type>, which can be GAB (Gabriel et al), GAM (4 gamete blocks), SPI (solid spine blocks) or ALL (each of the previous 3). The default block type is Gabriel. More information can be found with the blocks help. (See original Haploview Help file)

-blockCutHighCI <thresh>

Gabriel 'Strong LD' high confidence interval D' cutoff.

-blockCutLowCI <thresh>

Gabriel 'Strong LD' low confidence interval D' cutoff.

-blockMAFThresh <thresh>

Gabriel MAF threshold. Markers below this allele frequency will be skipped in building Gabriel blocks.

-blockRecHighCI <thresh>

Gabriel recombination high confidence interval D' cutoff.

-blockInformFrac <thresh>

Gabriel fraction of informative markers required to be in strong LD.

-block4GamCut <thresh>

4 Gamete block cutoff for frequency of 4th pairwise haplotype.

-blockSpineDP <thresh>

Solid Spine blocks D' cutoff for 'Strong LD'.

e) Other Output Options

-check

Output marker quality checks to <inputfile>.CHECK

-dprime

Output pairwise LD text table to <inputfile>.LD. Note that -dprime and -check default to no haplotype output unless the -blockoutput flag is also specified.

-png

Output PNG image file of LD display to <inputfile>.LD.PNG

-compressedpng

Output low-resolution (smaller file) PNG image of LD display to <inputfile>.LD.PNG

-spacing <threshold>

Use proportional spacing for dumped LD pngs. <threshold> ranges from 0 (no spacing) to 1 (max spacing) with a default of 0.

-ldcolorscheme <type>

Use a particular color scheme for dumped LD pngs. <type> can be DEFAULT, RSQ, DPALT, GAB or GAM. More information can be found with the LD display help (See original Haploview Help file).

-hapthresh <threshold>

Only output haplotypes with frequency ≥ <threshold>. Note that multiallelic D' and htSNPs are computed using only displayed haplotypes.

-excludeMarkers <markers>

Exclude markers in a comma separated list with ranges specified as start..end. So, to exclude markers 3, 5 and 10 through 15 you'd use "-excludeMarkers 3,5,10..15"

f) Tagger Output Options

-pairwiseTagging

Generates pairwise tagging information in <inputfile>.TAGS and .TESTS

-aggressiveTagging

As above but also allows 2- and 3-marker haplotype tags.

-maxNumTags <n>

Only selects <n> best tags.

-includeTags <markers>

Forces in a comma separated list of marker names as tags.

-includeTagsFile <file>

Forces in a file of one marker name per line as tags.

-excludeTags <markers>

Excludes a comma separated list of marker names from being used as tags.

-excludeTagsFile <file>

Excludes a file of one marker name per line from being used as tags.

-taglodcutoff <thresh>

Tagger LOD cutoff for creating multimarker tag haplotypes.

-tagrsqcutoff <thresh>

Tagger and/or Snagger r^2 cutoff.

g) Snagger Output Options

-snagger <outputDirectory>

Runs Snagger and outputs all relevant files to a subdirectory inside of the outputDirectory specified.

-scoreFile <file>

Specifies the Score File that contains SNP identifiers, chromosome, coordinate, strand, and design score.

-tagrsqcutoff <thresh>

Tagger and/or Snagger r^2 cutoff.

-includeTags <markers>

Forces in a comma separated list of marker names as tags.

-includeTagsFile <file>

Forces in a file of one marker name per line as tags.

-excludeTags <markers>

Excludes a comma separated list of marker names from being used as tags.

-excludeTagsFile <file>

Excludes a file of one marker name per line from being used as tags.

-noAffy

When this flag is specified, does not retrieve HapMap genotype data from the Affymetrix 500k Mapping Array.

-dataDir <dir>

Specifies a directory to retrieve HapMap files from. Each file must have the format: hapmap_{region name}_{population_id}.txt.
Examples:

hapmap_VDR_CEU.txt

hapmap_ESR1_CHB+JPT.txt

-region <regionName>

Specifies the region in the Score File for which data will be loaded. Required for use with Snagger.

-populations <populationList>

Specifies the HapMap population(s) for which data will be loaded. For Progressive Snagger, the population IDs should be separated by commas and listed in the desired tagging order. Combined population groups should be joined together with the '+' character and be listed in alphabetical order. Examples:

CEU

CEU,CHB,JPT,YRI

CEU,CHB+JPT,YRI

-surrogates <BINSIZE, PROBSUCC>

Adds surrogate tagSNPs using the 'bin size' or 'probability of success method'. Default settings will be used for each method unless specified with the theta1, theta2, minProbSucc, or minBinSize flags (see below).

-theta1 <value>

Specifies the *theta1* value for adding surrogates with the 'bin size' method. Default value is 1.0. See the Haploview with Snagger User Guide for details.

-theta2 <value>

Specifies the *theta2* value for adding surrogates with the 'bin size' method. Default value is 0.8. See the Haploview with Snagger User Guide for details.

-minProbSucc <threshold>

Specifies the minimum calculated probability of success (CPS) each marker should have when adding surrogates with the 'probability of success method'. Default is 0.999.

-minBinSize <threshold>

Specifies the minimum bin size for requiring a minimum calculated probability of success when adding surrogates with the 'probability of success' method. Default is 8.

-minScore <threshold>

Specifies the minimum design score for choosing tagSNPs. Value should be between 0 and 1.1. In order to exclude all SNPs below this threshold, you should use the –scoreExcludeAlways flag. Otherwise, by default, the SNPs below the threshold will excluded unless they become untaggable. Design scores of 0 are always excluded unless otherwise specified.

- dontExcludeScoreZero

Specifies that design scores of 0 should not always be excluded.

-scoreExcludeAlways

See –minScore flag.

-minTagDistance <0, 60>

The minimum distance between tagSNPs. Default is 60.

-probSuccWeight <value>

Advanced option. Changes the genotyping probability of success weighting (based on design score. Default is 1.0

-probSuccAlpha <value>

Advanced option. Alpha coefficient for calculating probability of success based from a design score. See 4)c)iv)(1)

-probSuccBeta <value>

Advanced option. Beta coefficient for calculating probability of success based from a design score. See 4)c)iv)(1)

-mafWeight <value>

Advanced option. Changes the MAF weighting. Default is 0.

-idealMaf <value>

Advanced option. Changes the ideal MAF to weight towards. Value must be between 0 and 0.5. Default is 0.5.

-codingStatusWeight <value>

Advanced option. Changes the coding status weighting. Default is 0.

-unknownCodingStatusScore <value>

Advanced option. Changes the score for unknown coding status. Value must be between 0 and 1. Default is 0.

-nonsynCodingStatusScore <value>

Advanced option. Changes the score for non-synonymous coding status. Value must be between 0 and 1. Default is 0.

-synonCodingStatusScore <value>

Advanced option. Changes the score for synonymous coding status. Value must be between 0 and 1. Default is 0.

-locusWeight <value>

Advanced option. Changes the locus weighting. Default is 0.

-score3UTR <value>

Advanced option. Changes the score for a locus value of ‘3UTR’. Value must be between 0 and 1. Default is 0.

-score5UTR <value>

Advanced option. Changes the score for a locus value of ‘5UTR’. Value must be between 0 and 1. Default is 0.

-scoreUTR <value>

Advanced option. Changes the score for a locus value of ‘UTR’. Value must be between 0 and 1. Default is 0.

-scoreCoding <value>

Advanced option. Changes the score for a locus value of ‘coding’. Value must be between 0 and 1. Default is 0.

-scoreFlanking3UTR <value>

Advanced option. Changes the score for a locus value of ‘flanking_3UTR’. Value must be between 0 and 1. Default is 0.

-scoreFlanking5UTR <value>

Advanced option. Changes the score for a locus value of ‘flanking_5UTR’. Value must be between 0 and 1. Default is 0.

-scoreIntron <value>

Advanced option. Changes the score for a locus value of ‘intron. Value must be between 0 and 1. Default is 0.

-scoreUnknownLocus <value>

Advanced option. Changes the score for a locus value of ‘-1’. Value must be between 0 and 1. Default is 0.
